# Telemedical percussion: objectifying a fundamental clinical examination technique for telemedicine

**DOI:** 10.1007/s11548-021-02520-z

**Published:** 2021-11-24

**Authors:** Roman Krumpholz, Jonas Fuchtmann, Maximilian Berlet, Annika Hangleiter, Daniel Ostler, Hubertus Feussner, Dirk Wilhelm

**Affiliations:** 1 Research Group MITI - Minimally Invasive Interdisciplinary Therapeutical Intervention, Technical University Munich, Munich, Germany; 2 Department of Surgery, Klinikum rechts der Isar, Technical University Munich, Munich, Germany

**Keywords:** Percussion, Robotic examination, Robotic percussion, Telemedicine, Tele-diagnostic, Auscultation

## Abstract

**Purpose:**

While demand for telemedicine is increasing, patients are currently restricted to tele-consultation for the most part. Fundamental diagnostics like the percussion still require the in person expertize of a physician. To meet today’s challenges, a transformation of the manual percussion into a standardized, digital version, ready for telemedical execution is required.

**Methods:**

In conjunction with a comprehensive telemedical diagnostic system, in which patients can get examined by a *remote-physician*, a series of three robotic end-effectors for mechanical percussion were developed. Comprising a motor, a magnetic and a pneumatic-based version, the devices strike a pleximeter to perform the percussion. Emitted sounds were captured using a microphone-equipped stethoscope. The 84 recordings were further integrated into a survey in order to classify lung and non-lung samples.

**Results:**

The study with 21 participants comprised physicians, medical students and non-medical-related raters in equal parts. With 71.4% correctly classified samples, the ventral motorized device prevailed. While the result is significantly better compared to a manual or pneumatic percussion in this very setup, it only has a small edge over the magnetic devices. In addition, for all ventral versions non-lung regions were rather correctly identified than lung regions.

**Conclusion:**

The overall setup proves the feasibility of a telemedical percussion. Despite the fact, that produced sounds differ compared to today’s manual technique, the study shows that a standardized mechanical percussion has the potential to improve the gold standard’s accuracy. While further extensive medical evaluation is yet to come, the system paves the way for future uncompromised remote examinations.

## Introduction

Percussion has a long history in medicine. Dating back to the fifth-century BC, the Greeks used a technique called “hippocratic succussion” to detect free gas or fluid inside a patients body. By shaking the patient, observable splashing noises were created [15]. In modern Medicine, Leopold Auenbrugger introduced 1763 the “percussion’ to distinguish between empty and filled body cavities. The method involved tapping on the patient’s body surface in an affected region in order to produce sounds of resonance [1].

**Fig. 1:**
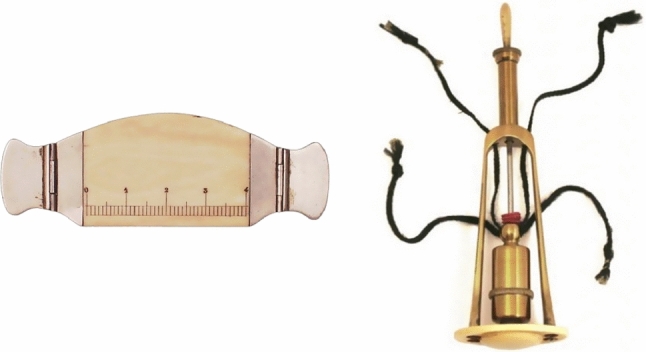
Left: Ivory pelximeter with handles used to transfer the strikes during a percussion onto the patient’s tissue [8]. Right: Sibson’s percussor-pleximeter, a percussion device with an integrated pleximeter, allowing for higher standardized diagnostics [8]

Since then, popularity of percussion in medicine increased and it became a fundamental part of clinical examination and is still frequently used today. It allows the physician to determine the size of organs and therefore can support the diagnosis of pathologies like lung emphysema, pneumothorax, enlarged abdominal organs or fluid accumulation. In 1826, Pierre Adolphe Piorry proposed the pleximeter, an ivory pad placed between the tapping finger and the patient’s body surface; see left image of Fig. 1. However, the technique did not prevail, as fingers of the second hand were used more commonly instead of the pleximeter, facilitating the procedure [23].

Over time, multiple approaches tried to objectify the diagnostic method, as results of the percussion mainly rely on the individual skills and expertize of the physician. While the execution itself is not standardized, interpretation of the resulting sound is also only based upon previously gained experience [6, 18]. Furthermore, results cannot be captured and presented to colleagues or compared to previous examinations. Already in the 1850s, Francis Sibson developed a percussion-pleximeter, depicted on the right image in Fig. 1. The brass cylinder is attached to rubber bands and the fixed movement was mend to improve reproducibility [8]. In 1895, Cartex tried to visualize the acoustic resonance wave with a membrane, thereby controlling the gas supply of a flame. Other visualization and measuring techniques relied on taping the signal to wax-based cylinders. Ultimately, the manual percussion without any assistive equipment is established today. [13].

Only recently, multiple new IoT devices have been developed and not least because of the COVID-19 pandemic, demand for telemedicine increased noticeably [14]. Devices such as the Tyto Home (Tyto Care Inc., Netanya, Israel) provide a portable solutions to capture basic vital signs and provide them remotely to a physician. However, current available equipment is limited to a small set of parameters and full reproduction of an in person examination in telemedicine is not available [7]. Regarding an automatic percussion, only few devices have been proposed yet [2, 3, 19, 21]. While Peng and Dai [19] used a permanent magnet shaker, Ayodele and Ogunlade [2] applied a push-pull solenoid plessor for the percussion generation. Rao and Ruiz [21] utilized a neodymium audio driver and Bohadana and Kraman [3] assessed a pneumatic cylinder. While this paper builds upon findings in literature, it also considers alternative, previously unused techniques to generate suitable impulses. Moreover, it considers the specific requirements regarding a telemedical execution of the percussion and tries to overcome shortcomings of previous systems.

## Methodology

In the following paragraph, the framework in which the development of the telemedical percussion devices was carried out will be presented first. Afterwards, the different versions of the percussion instruments are described as well as the experimental setup used for evaluation of the percussion instruments.

### Telemedical diagnostic system

**Fig. 2:**
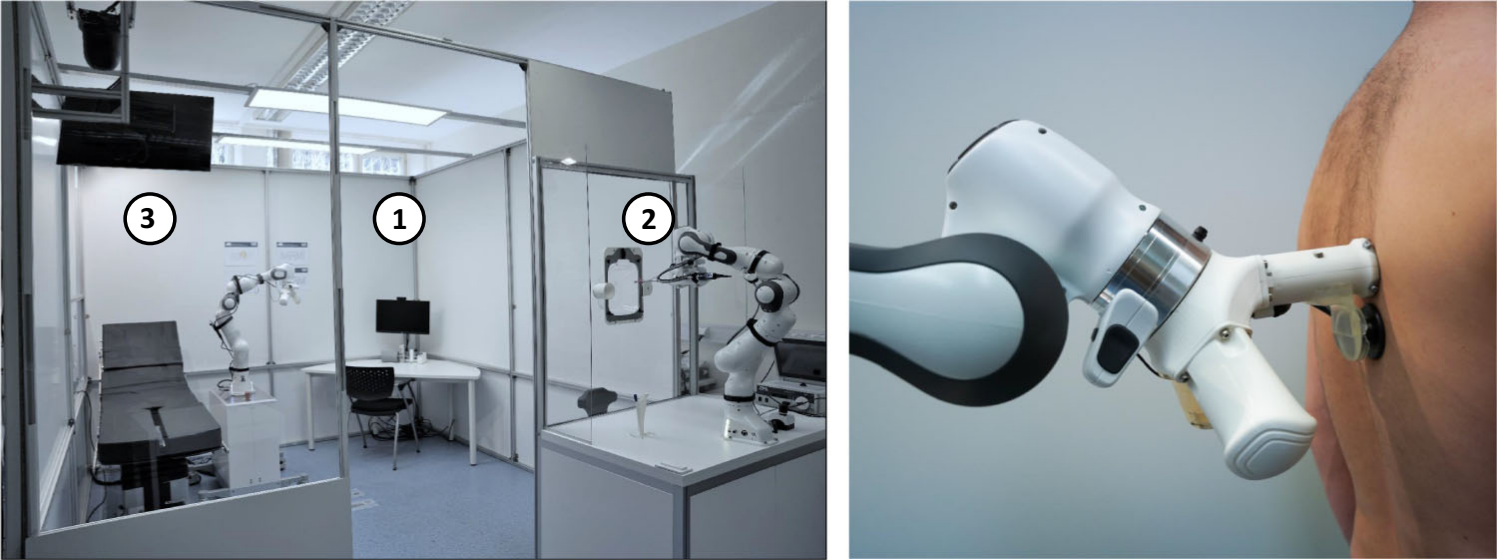
Left: Photograph of the examination cabin. White circles indicate the distinct examination modules. Right: Detail shot, showing the revolver-like fixture at the robotic arm of module 3 with the attached palpation and auscultation device, as well as the magnetic percussion end-effector during evaluation

In order to expand current possibilities in telemedicine, a comprehensive tele-diagnostic system was developed [9]. The system is integrated into a special cabin as shown in Fig. 2. When a patient enters the cabin, he can remotely be assessed by a physician using a sophisticated set of tele-diagnostic devices. The cabin includes three major modules.

Module one consists of a desk equipped with an display-camera combination, an otoscope, a thermometer, a blood pressure cuff and a device for measurement of the blood oxygen saturation (pulse oximetry). Here the physician can obtain the chief complaint and gather information such as the vital signs.

In module two, a *Panda* robotic arm (Franka Emika, Munich, Germany) is used to collect a mouth and nose swab sample, as well as to perform an inspection of the oral cavity. The robot can therefore switch between a videoendoscope *FIVE S* (Karl Storz, Tuttlingen, Germany) and a gripper for the swab. The robotic arm and the patient are thereby physically separated using an acrylic glass pane. Small openings allow only the tip of the endoscope or swab to be introduced into the oral or nasal cavity.

The key component of the third module is a further *Panda* robotic arm. As depicted in Fig. 2, a revolver-like adapter at the robotic arms end enables up to three individual end-effectors to be used for examination. Additional to a straightforward palpation end-effector, a stethoscope was mounted on a flexible fixture and a heavy duty microphone of the *4660 series* (DPA Microphones, Alleroed, Denmark) was integrated. Thus, audio signals of an auscultation can be digitally transferred to the remote physician. The third mounting plate offers an attachment possibility for the percussion devices discussed in “Percussion instruments” section. Hence, an auscultation, percussion or palpation is feasible while the patient is standing upright or lying on the examination couch. Furthermore, a blood sample can optionally be taken from assistive personal outside the cabin via a protected transfer hatch next to the examination couch.

On the physician’s remote desk, a proprietary developed interface provides access to all information gathered by the diagnostic devices. For an optimal view, multiple cameras, including high-resolution pan-tiled-zoom cameras pave the way for a seamless patient-physician interaction. An additional *Panda* robotic arm serves as a remote controller for the ones located at the patients side. Movements and force applied on the joints of the robots are directly transferred in both directions. While safety limits regarding speed, acceleration and positions are predefined, the system provides force feedback and thereby increases safety precautions.

Apart from executing a palpation and an auscultation, the robotic system of module 3 is especially important regarding realization of a proper percussion. Precise positioning of the end-effectors is crucial for obtaining meaningful results. Hence, examinations have to be conducted by physicians them-self or with respect to a telemedical execution, by a robotic system. Use of the setup as previously described thereby implies some boundary conditions regarding the implementation. First and foremost, weight of all end-effectors is restricted to the total load capacity of the robotic head. In case of the *Panda* platform, total pay load is 3 kg. Considering previous solutions, such as the one used by Peng and Dai [19] the percussion devices alone can weigh more than 2.7 kg. Furthermore, a small construction size is preferable to avoid interference with the other end-effectors.

Previous publications introducing mechanical devices [2, 3, 19, 21] only tested their constructions for a ventral percussion, whereby patients had to hold devices themselves. Even though Guarino [10] presented the technique of ventral sternal chest percussion with a simultaneous dorsal auscultation in his original publication as efficient, later studies questioned this technique [4, 5, 16]. Since today’s average manual chest percussion is also performed on the patient’s back, a further evaluation of automatic dorsal percussion is required. Moreover, a solution independent on a combination of percussion and auscultation on opposing sides would reduce complexity to one combined robotic end-effector, voiding the patient’s handheld device.

### Percussion instruments

For an initial approach to robotic percussion, 3 percussion instruments were developed, differing in their mode of operation:

- Motorized (MOT)
- Magnetized (MAGN)
- Pneumatic (PNEU)

As gold standard, the manual percussion using the finger tips was applied for comparison:

- Manual (MAN)

To ensure the percussion quality of the instruments for all types of configuration found in the literature, both a ventral (patient’s front) and a dorsal (patient’s back) version were developed for each instrument. In the following, the capital letter ’V’ or ’D’ represents the body side the percussion is applied to:

- Ventral (-V)
- Dorsal (-D)

The dorsal version combines the function of percussion and auscultation in one device. During the examination, the device is guided solely by the robot, so the patient does not have to perform any task. The ventral version, on the other hand, has a separate adapter for percussion and auscultation. During the examination, the percussion adapter is pressed onto the patient’s sternum by the patient themselves, while the robot places the auscultation adapter on the patient’s back. The ventral and dorsal version of an end-effector differ only structurally, not functionally. The dorsal versions provide an additional structure that is used for fixing the stethoscope. The ventral version, instead, do not have this structure. In the following, the ventral versions of the percussion adapters are presented. As a representative for the dorsal version, the magnetic percussion instrument is shown in Fig. 2b.

#### General setting

In contrast to the previous approaches of [3, 19, 21], percussion sounds were picked up over the patient’s body surface. Instead of using a room microphone to record percussion sounds, a stethoscope was modified using a heavy duty microphone of the *4660 series* (DPA Microphones, Alleroed, Denmark). Reason for that decision is the interfering noise prevailing from the instrument and the *Panda* arm during operation.

The material combination between the percussion head and the pleximeter is identical for all instruments. The percussion head consists of a 3D-printed bracket in which a rubber nipple of a percussion hammer (DocCheck Community GmbH, Cologne, Germany) is form-fitted held in position. This prevents both axial and radial movement. Via a threaded coupling, the percussion head can be mounted on the respective instruments. Spring rings prevent the head from becoming loose during the percussion process and resulting vibrations. The pleximeter (H. Hauptner und Richard Herberholz GmbH & Co. KG, Herberholz, Solingen, Germany) consists of a 3-mm-thick, spatula-shaped plate which is pressed firmly onto the spot of the body to be examined.

#### Motorized percussion

**Fig. 3:**
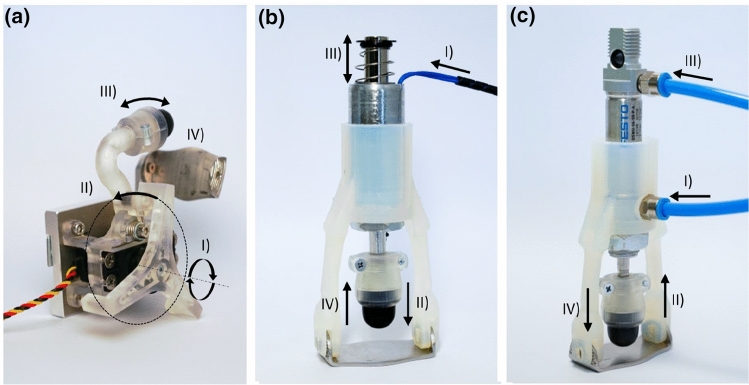
Inner workings of the three different percussion instruments. Picture **a** shows the spring-loaded motorized version, picture **b** depicts the transistor controlled solenoid and picture **c** displays the pneumatic percussion instrument, driven by an external supply of compressed air

As shown in Fig. 3a, the driving unit of the motorized end-effector (MOT) is an *HSR-2645 CR* continuous drive servo motor (HITEC, Sand Diego, USA). A transmission rotor (Fig. 3a I), which is mounted on the outlet shaft of the servo motor, engages with an impact element via a spur gear (Fig. 3a II). The striking element is rotatably mounted in the housing of the adapter by means of a torsion spring. The percussion head is located at the end of the striking element. The spring allows the striking element to be loaded so that the energy is stored during engagement (Fig. 3a III). As soon as the engagement between rotor and striking element ends, the restoring force of the spring causes the percussion head to strike the pleximeter (Fig. 3a IV). The number of spokes $n_{\mathrm{Spoke}}=3$ of the rotor and the rotation speed $n_{\mathrm{Mot}}=72$ RPM of the servo motor results in a defined impact frequency of 3.6 Hz. The adapter is controlled via an *Atmega2560* (Atmel, San José, United States), which specifies the speed of the servo motor via a PWM signal. The selected spring has a maximum torque of $M_{\mathrm{Spring}}=357$ Nmm. The impact element has a length of 20 mm, resulting in $F_{\mathrm{Impact}}=17.85$ N. The duration of the impulse is defined by the bearing of the impact element. With the help of the elastic bearing, the striking element springs back after hitting the pleximeter. The mechanism is acoustically damped by an insulated enclosure.

#### Magnetized percussion

The magnetic percussion instrument’s (MAGN) drive unit, shown in Fig. 3b, is an *ITS-LZ 2560-D-12VDC* solenoid (Intertec Components, Freising, Germany). The solenoid is mounted in a cylindrical housing to prevent both axial and radial movements. The percussion head is screwed onto the anchor of the solenoid with the help of a connection sleeve. The pleximeter is fixed within provided cavities in the housing of the adapter. With the help of slotted holes, the pleximeter is aligned according to the stroke of the magnet. The solenoid is controlled by means of a transistor circuit. An Atmega2560 determines the frequency of the percussion. When the transistor is switched through, current flows into the solenoid (Fig. 3b I), pulling the core within the coil carrier (Fig. 3b II) and compressing the spring, placed at the top of the core, between the core and the coil (Fig. 3b III). As soon as the transistor blocks, the spring returns the solenoid to its original position (Fig. 3b IV). The relative duty cycle of 125 ms results in an impact frequency of $f_{\text {Impact, Magnetic}}=4$ Hz. With a stroke of 7.5 mm, the solenoid characteristic leads to an impact force of $F_{\text {Impact, Magnetic}}=19$ N. The duration of the impulse does not approach zero compared to the motorized adapter. The comparatively weak spring is responsible for this, why the action depends primarily on the fine adjustment between the impact head and the pleximeter. Acoustic insulation is not necessary due to the low noise level of the solenoid (Table 1).

**Table 1:** Overview of the key parameters of the developed percussion instruments

Percussion instruments	Frequency (Hz)	Force (N)	Stroke (mm)
Motorized percussion (MOT)	3.6	17.85	116.8
Magnetic percussion (MAGN)	4	19	7.5
Pneumatic Percussion (PNEU)	4	20	10

In order to create a comparable setup for all instrument versions, same percussion rate and impact force were chosen

#### Pneumatic percussion

The pneumatic percussion instrument (PNEU) as shown in Fig. 3c has a *19198 DSNU-16-10-P-A* round cylinder (Festo, Esslingen, Germany) installed as the drive unit. The double-acting cylinder is mounted in an enclosure, similar to the solenoid. Both axial and radial slippage of the cylinder relative to the housing are prevented. The percussion head is mounted on the piston rod of the cylinder with the help of a threaded sleeve. The pleximeter is mounted on two extension arms. Elongated holes allow the distance between the pleximeter and the percussion head to be adjusted. Using two pneumatic pipes, the adapter is connected to a *3V210-08* 3/2 way solenoid valve (Heschen, Zhejiang, China). The valve is controlled by an *Atmega2560*, which allows the impact frequency of the adapter to be defined. The relative duty cycle of 125 ms results in a stroke rate of $f_{\text {Impact, Pneumatic}}=4$ Hz. With a stroke of 10 mm, an impact force of $F_{\text {Impact, Pneumatic}}=20$ N results from the piston diameter and the operating pressure of $p_{\text {Impact, Pneumatic}}=1$ bar. Due to the double-acting mode of operation, no free return movement of the piston is possible after the impact on the pleximeter. Thus, the duration of impact is once again dependent on the fine adjustment of the distance between the impact element and the pleximeter. Once the front chamber of the cylinder is actuated (Fig. 3c I) by the valve, the percussion head is pushed in (Fig. 3c II). Depending on the switching time of the valve, the rear chamber is actuated next (Fig. 3c III), which causes the percussion head to accelerate onto the pleximeter (Fig. 3c IV). In operation, the pneumatic adapter is noisy compared to the motorized and magnetized end-effectors due to the venting of the cylinder. This is remedied by an insulated box in which the solenoid valve is located and with silencers at the exhaust outlets of the solenoid valve.

### Experimental setup

In order to implement the best percussion device for the telemedical diagnostic setup described in “Telemedical diagnostic system” section, the three particular instruments were evaluated in two different procedures. In procedure one, dorsal versions of the devices (-D) were placed on a healthy volunteer’s back. In procedure two, the ventral percussion instruments (-V) were embedded into handheld units, which were placed on the sternum by the test person himself. Auscultations were then executed by the robotic arm on the back side. Since the stethoscope is mounted to the robotic arm in both procedure types, a physician always has complete flexibility regarding the spots to be examined.

**Fig. 4:**
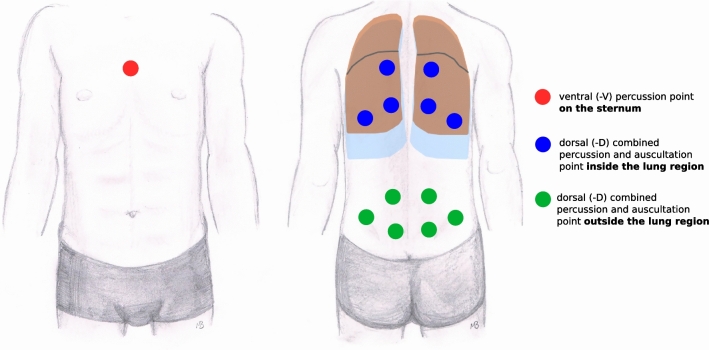
Percussion points on the ventral (left) and dorsal (right) patient side. The lung region is highlighted. Auscultation was always carried out dosal and in close proximity to the percussion end-effector

Signals captured by the microphone were converted into 24-bit/192 kHz digital recordings using a *UR824* interface (Steinberg Media Technologies GmbH, Hamburg, Germany). All recordings were used without any further signal alteration. For each percussion device and each procedure, 6 spots within and 6 spots outside of the pulmonary area were measured. Points are depicted in Fig. 4. Thereby two of the samples for each combination were presented to raters as annotated reference sounds. In order to reference the outcome to the gold standard, a manual percussion was performed and captured as well.

All 84 samples were integrated into a survey. Samples were grouped per method and device within a video and hosted on a server for an online poll using the software *LimeSurvey* version 3.25.3 + 201208. Grouping samples for each device allow for a comparative evaluation within each sample set and thus resemble today’s manual method where the attenuation is always assessed relatively in relation to other auscultation spots.

Trained medical staff ranging from senior consultant level to medical students and non-medical participants were then asked to label each sample with the options lung, non-lung and no answer. Furthermore, they were asked about the output audio device used for the survey. Participants were not informed about the data balance of lung and non-lung samples and had to choose individually for each new sample. However, for each new device version and percussion method, reference audio clips with known labels were provided. Results were analyzed using the statistical software *R* version 3.6.3 without additional packages [20]. Because of the discrete ordinal scale of the data, non-parametric multivariate statistical methods such as the Friedman–Wallis test with post-hoc Wilcoxon rank sum test were chosen to reveal statistical significance. A significance level of 5% was set.

## Results

A total of 21 people participated in the online study. Table 2 displays the participant’s baseline characteristics. The group of test-persons was equally balanced regarding multiple levels of medical expertize and comprised 6 fully trained medical doctors, 6 medical students and 6 attendees without previous knowledge. Participants had free choice regarding the used output device. 19.0% used in-ear headphones, 33.3% classic headphones and 47.6% the in-built speakers of the respective device.

**Table 2:** Baseline characteristics of the participants

			In-ear headphones	Classic headphones	Device speakers
	%		23.8	28.5	47.6
		*n*	5	6	10
Medical student	33.3	7	2	1	4
Medical doctor (MD)	33.3	7	1	2	4
Other	33.3	7	2	3	2

*n*, absolute frequency; %, relative frequency

**Fig. 5:**
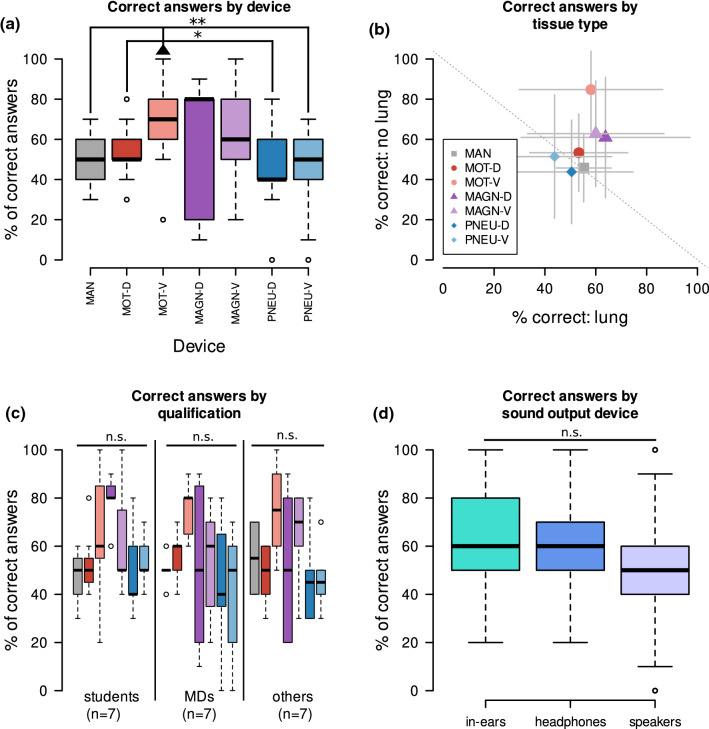
Device abbreviations—*MAN* manual percussion, *MOT-D* mechanic device with dorsal percussion and auscultation, *MOT-V* mechanic device with ventral percussion and dorsal auscultation, *MAGN-D* magnetic device with dorsal percussion and auscultation, *MAGN-V* magnetic device with ventral percussion and dorsal auscultation, *PNEU-D* pneumatic device with dorsal percussion and auscultation, *PNEU-V* pneumatic device with ventral percussion and dorsal auscultation, the color scheme from **A** and **B** is also used in **C**, in **B** the symbols mark the mean and the whiskers the standard deviation on the respective axis/**p value < 0.01, *p value < 0.05, *n.s.* *p* value $\ge$ 0.05 in Friedman test with Wilcoxon rank sum test as post-hoc analysis, *p* values are adjusted by Bonferroni correction

In the overall group, there were 3 favorite instruments found. The use of the motorized device with ventral percussion (MOT-V) was associated with a significantly better rate of correct assignment of the torso part than the motorized device with dorsal percussion (MOT-D), manual percussion (MAN) and both pneumatic devices (PNEU-D and PNEU-V). As depicted in Fig. 5A, the two magnetic versions (MAGN-D and MAGN-V) even had the tendency of a better rate of correct answers, however without statistical significance.

To gain a more comprehensive characteristic, we analyzed rates of correct assignments of a sound and auscultation spot for each single device and auscultation procedure. Results are displayed in a dotplot with the percentage of correct lung classification rate on the *x*-axis and the non-lung classification rate on the *y*-axis. Recognition of non-lung tissue was commonly better than the one of lung. While the device MOT-V reached a mean of more than 80%, Fig. 5B, regarding recognition of non-lung tissue, the average of 75% was not exceeded by any device for the recognition of lung tissue.

**Table 3:** Correct answers by qualification and device

Group	MAN	MOT-D	MOT-V	MAGN-D	MAGN-V	PNEU-D	PNEU-V
*Medical students* $(n=7)$							
Mean (SD)	47.1	52.9	65.7	80.0	62.9	50.0	54.3
	(11.1)	(13.8)	(28.2)	(10.0)	(22.9)	(18.3)	(9.8)
Median (IQR)	50.0	50.0	60.0	80.0	50.0	40.0	50.0
	(15.0)	(10.0)	(30.0)	(5.0)	(25.0)	(20.0)	(10.0)
*Medical doctos* $(n=7)$							
Mean (SD)	50.0	55.7	74.3	51.4	52.9	45.7	40.0
	(5.8)	(9.8)	(11.3)	(35.3)	(24.3)	(27.0)	(27.1)
Median (IQR)	50.0	60.0	80.0	50.0	60.0	40.0	50.0
	(0.0)	(10.0)	(15.0)	(65.0)	(35.0)	(30.0)	(40.0)
*Others* $(n=7)$							
Mean (SD)	55.0	48.3	75.0	51.7	65.0	46.7	46.7
	(13.8)	(11.7)	(18.7)	(34.9)	(19.7)	(18.6)	(13.7)
Median (IQR)	55.0	50.0	75.0	50.0	70.0	45.0	45.0
	(25.0)	(15.0)	(25.0)	(60.0)	(20.0)	(17.5)	(10.0)
*All* $(n=21)$							
Mean (SD)	50.5*	53.3*	71.4*	62.4	61.4	47.1*	47.6*
	(10.2)*	(12.0)*	(19.6*)	(30.2)	(22.4)	(20.3)*	(18.4)*
Median (IQR)	50.0*	50.0*	70.0*	80.0	60.0	40.0*	50.0*
	(20.0)*	(10.0)*	(20.0)*	(60.0)	(30.0)	(20.0)*	(20.0)*

Numbers represent percentages (%) of correct answers

Device abbreviations—*MAN* manual percussion, *MOT-D* mechanic device with dorsal percussion and auscultation, *MOT-V* mechanic device with ventral percussion and dorsal auscultation, *MAGN-D* magnetic device with dorsal percussion and auscultation, *MAGN-V* magnetic device with ventral percussion and dorsal auscultation, *PNEU-D* pneumatic device with dorsal percussion and auscultation, *PNEU-V* pneumatic device with ventral percussion and dorsal auscultation, *SD* standard deviation, *IQR* interquartile range

The bold printed results differ statistically significantly from the others marked with an asterisk (*)

As shown in Fig. 5C, stratification by the participant’s qualification even revealed a tendency toward the three mentioned favorites, however without statistical significance in the non-parametric testing.

Even the kind of sound output used during the online study, did not influence the percentage of correct assignment. Although there was a tendency toward a better median sum of correct answers in the group of participants using headphones, this observation was not statistically significant; see Fig. 5D.

## Discussion

To investigate the feasibility of a robotic percussion, three different instruments were developed. Each of the functionalities has proven its viability, but also specific disadvantages during operation have been observed.

According to the study results, the highest probability of correctly identified samples is for the device MOT-V with motorized ventral percussion. Despite the superiority of the instrument, the gearbox of the servo motor used generates non-negligible noise that affects the audio signal analyzed by the physician. For further developments, the usage of low-noise geared motors is imperative.

The magnetic ventral percussion instrument ranks directly after the motorized version according to the study results. Due to its comparatively quiet mode of operation, the solenoid generates hardly any noticeable ambient noise. Its very compact design also favors its usage as a robotic instrument. However, from a medical perspective the magnetic function mechanism may bear the risk of interfering with a patient’s pacemaker or other implants [12]. Therefore, frequency of the electromagnetic field generated by the solenoid should be taken into account in subsequent developments (Table 3).

In comparison, the lowest performance was shown by the pneumatic percussion instrument. One reason therefor could be the double-acting mode of operation of the cylinder. After the percussion adapter hits the pleximeter, no free return movement by the head is possible. This results in a comparatively long pulse duration. Another disadvantage of the pneumatic cylinder is the pneumatic tube. Disconnection or bending can completely disrupt the adapter, making the intervention of an assistant unavoidable. In addition, the solenoid valve used requires acoustic insulation, which results in a trade-off. Either the valve is positioned close to the percussion and ambient noise is accepted despite insulation, or the solenoid valve is positioned as far away from the patient as possible. The latter, however, leads to an increased tube length, which requires a drastically higher working pressure.

According to the study results, it can be observed that the output medium used has a tendency to influence the quality of the percussion sound from the user’s side. Although this was not statistically significant, a cautious recommendation can be made for the use of (in-ear) headphones. Having said this, participants may have used smartphone speakers during the poll and the category of “speakers” is not directly linked to professional HiFi loudspeakers.

Overall performance of the system may further be diminished compared to a non-robotic percussion, as navigation between different auscultation points is more time consuming compared to in person interaction. This increases time intervals between the sample capturing and obstructs the comparing auditory analysis. Across all participants, the ventral versions of motorized percussion showed the least variation. However, from a technical point of view, the dorsal percussion adapters would be preferable as they combine the function of percussion and auscultation in one device. This would make percussion completely feasible only with a robotic arm and without the patient’s cooperation. However, at all times the medical quality of percussion has to be at the forefront of the development for a robotic variant. Subsequent developments should therefore be designed for ventral percussion, even though co-operation with the patient or implementation of modified robotic percussion adapter is required.

Apart from the quantitative study results, multiple raters reached out and marked that the mechanical percussion sounds fundamentally different compared to what they are used to. However, this feedback was expected, as the primary goal was never to acoustically mimic the manual percussion. Forthcoming telemedical systems will generally change the way diagnostic data is presented and whether virtual emulation can provide benefits remains to be seen. Further, this study did not asses the patients acceptance of a mechanical system executing the already manually rather unpleasant examination.

## Conclusion

To meet requirements of a future telemedical assessment of patients, we developed a series of three different mechanical percussion devices. The devices comprise a motor-spring based, a pneumatic-based as well as a magnetic-based version and are used in conjunction with a pleximeter to strike the patient’s surface equivalently to a manual percussion. While acoustic signals are captured by a microphone equipped stethoscope, the overall instrumentation is compatible with a robotic-based telediagnostic setup. Thus flexibility regarding the patient individual positioning, coming along with the physicians expertize is maintained.

In a study with 21 raters, motorized ventral percussion performed best, closely followed by the ventral and dorsal magnetic percussion. The trade-off regarding usability and medical aspects such as interference with implants has to be weighed well. While this proof of concept was designed to determine the best technical approach for a robotic percussion, a major next step will be the evaluation of the ventral motorized instrument regarding detection of different pathologies. Furthermore, different areas of medicine already use automatized analysis, such as deep learning approaches, for medical audio signals [2, 11, 17, 22].

## Data Availability

The used survey form and individual results can be provided on request.
